# Rapid and Stable Formation Method of Human Astrocyte Spheroid in a High Viscous Methylcellulose Medium and Its Functional Advantages

**DOI:** 10.3390/bioengineering10030349

**Published:** 2023-03-11

**Authors:** Fumiya Tao, Keita Kitamura, Sanshiro Hanada, Kazuyuki Sugimoto, Tomomi Furihata, Nobuhiko Kojima

**Affiliations:** 1 Department of Life and Environmental System Science, Graduate School of Nanobioscience, Yokohama City University, 22-2 Seto, Kanazawa-ku, Yokohama, Kanagawa 236-0027, Japan; 2 Laboratory of Clinical Pharmacy and Experimental Therapeutics, School of Pharmacy, Tokyo University of Pharmacy and Life Sciences, 1432-1 Horinouchi, Hachioji, Tokyo 192-0355, Japan; 3 Yokogawa Electric Corp., 2-3, Hokuyodai, Kanazawa, Ishikawa 920-0177, Japan

**Keywords:** astrocytes, cell immortalization, differentiation status, spheroid formation method

## Abstract

Astrocytes, a type of glial cell in the brain, are thought to be functionally and morphologically diverse cells that regulate brain homeostasis. Cell immortalization is a promising technique for the propagation of primary human astrocytes. The immortalized cells retain their astrocytic marker mRNA expression at lower levels than the primary cells. Therefore, improvement of the differentiation status is required. The use of a 3D formation technique to mimic structural tissue is a good strategy for reflecting physiological cell–cell interactions. Previously, we developed a spheroid formation method using highly viscous methyl cellulose (MC) medium. In this study, we applied this formation method to the well-established immortalized human astrocyte cell line HASTR/ci35. Stable HASTR/ci35 spheroids were successfully formed in MC medium, and laminin deposition was detected inside of the spheroids. Their functional markers were enhanced compared to conventional spheroids formed in U-bottom plates. The inflammatory response was moderately sensitive, and the ability to support neurite growth was confirmed. The HASTR/ci35 spheroid in the MC medium demonstrated the differentiation phenotype and could serve as a potent in vitro model for matured astrocytes.

## 1. Introduction

Astrocytes, a type of glial cells in the brain, are thought to not only support cells for neural function or the blood–brain barrier integrity but also lead cells to regulate synapse formation, function, and pruning [1,2]. As a cellular component in the tripartite synapse, astrocytes exert clearance and recycle various neurotransmitters, such as glutamate and gamma-aminobutyric acid (GABA) [3,4], and supply energy metabolites, lactate to neurons [5,6,7].

Although physiologically and pathologically relevant in vitro model cells are required to investigate human astrocytes, the isolation and propagation of primary human astrocytes is technically limited. Several research groups have tried to generate human astrocyte lineage cells derived from human pluripotent stem cells [8,9]. Sloan et al. reported that the long-term cultivation of human cortical spheroids enabled the purification of astrocyte lineage cells. However, as astrocytes are functionally and morphologically diverse cells [10,11,12], the unidirectional differentiation of astrocytes is still under development. For this purpose, cell immortalization is a promising technique because both infinite proliferation capacity and differentiation phenotypes can be simultaneously acquired in immortalized cells. In a previous report, Furihata et al. established conditionally immortalized human primary astrocytes (Human ASTRocyte/Conditionally Immortalized, clone 35: HASTR/ci35), where the temperature-sensitive simian virus 40 large T antigen (tsSV40T) gene and human telomerase catalytic subunit (hTERT) gene were introduced [13]. These HASTR/ci35 cells show proliferation at 33 °C owing to the temperature-sensitive SV40T. The growth is, however, inhibited under the culture condition at 37 °C and fetal bovine serum (FBS)-free, and the cells show relatively differentiated properties [14].

HASTR/ci35 cells retain their astrocytic marker mRNA expression at lower levels than the primary cells. The aim of this study is trying to improve the differentiation status of the immortalized cells. One of the strategies to enhance maturation of the cells is the 3D culture technique to mimic the structural tissue and to reflect on physiological cell–cell interactions. Urich et al. reported that 3D spheroids of neurovascular units could reconstitute the morphological characteristics by combining human primary pericytes, astrocytes, and microvascular endothelial cells [15]. However, 3D formation of astrocytes alone has not been realized because astrocytes usually lack the ability to form cell–cell interfaces. It means that there is no adequate method to form spheroid with HASTR/ci35 cells.

In this study, we attempted to enhance the function of the immortalized astrocyte cell line using our original 3D spheroid formation method with highly viscous methyl cellulose (MC) culture medium [16,17]. This compaction method enables the formation of cell aggregates even the cells have relatively lower cell–cell adhesion properties [18,19]. Aggregation of cells might increase cell differentiation status by increasing of cell–cell interactions. The astrocytic spheroids were analyzed from the aspect of differentiation state, inflammatory reaction, and neurite growth support.

## 2. Materials and Methods

### 2.1. HASTR/ci35 Cells

Establishment of HASTR/ci35 cells was previously reported by Furihata et al. [13]. The cells were grown at 33 °C in Dulbecco’s modified Eagle’s medium (DMEM; FUJIFILM Wako, Osaka, Japan) supplemented with GlutaMAX-I (Thermos Fisher Scientific, Waltham, MA, USA), 1% N-2 Supplement (FUJIFILM Wako), 10% (*v*/*v*) fetal bovine serum (FBS; Biowest, Nuaillé, France), penicillin-streptomycin (FUJIFILM Wako), and 4 µg/mL blasticidin S (FUJIFILM Wako) to maintain immortality. For the differentiation of astrocytes, the cells were cultivated at 37 °C in a medium without FBS. Since the cells were established from commercially available cells, no ethical considerations were required.

### 2.2. MC Medium

A 3 g aliquot of MC (viscosity, 4000 cP; M0512; Sigma-Aldrich, St. Louis, MO, USA) was placed in a glass bottle and sterilized by autoclaving. To the MC, 100 mL of DMEM (FBS free) was added (final concentration, 3% *w*/*v*), and the mixture was stirred with a magnetic stirrer in a cold room until the MC was completely dissolved. Viscous MC medium (2 mL) was dispensed into a 35 mm culture dish using a positive-displacement pipette (Gilson, Middleton, WI, USA).

### 2.3. Spheroid Formation

Spheroid formation using our unique method has been previously reported [16,17]. Briefly, 1 µL of the cell suspension (6000 cells) was serially injected into the MC medium using an electronic micropipette (Eppendorf, Hamburg, Germany). Up to 100 spheroids were able to be formed in a 35 mm dish. After injection, the culture dish was incubated at 37 °C under 5% CO_2_ until spheroids formed in 2–3 days. To collect the spheroids from the MC medium, 0.1 g/mL cellulase (Onozuka RS; Yakult Pharmaceutical Industry, Tokyo, Japan) in phosphate-buffered saline (FUJIFILM Wako) was added onto the MC medium, and the culture dish was incubated for 60 min at 37 °C. This digestion step enabled the efficient collection of spheroids in a sample tube. Two conventional spheroid formation methods (hanging drop method and low-cell-attachment U-bottom plate method) were examined simultaneously for benchmark experiments. The cells were cultured for 2–3 days using each method. The number of cells per spheroid was determined considering that the spheroids should have a size that is easy to handle and that troubles such as internal necrosis should not occur.

### 2.4. Spheroid Histological Staining

Paraffin sections of spheroids were deparaffinized and immersed in 10 mM citrate buffer (pH 6.0; FUJIFILM Wako), followed by autoclaving at 121 °C for 10 min. The sections were incubated with primary antibodies against laminin (L9393; Sigma-Aldrich), stained with Alexa 568-conjugated secondary antibodies, and counterstained with Hoechst 33,342 (Dojindo Laboratories, Kumamoto, Japan). The paraffin sections were stained with hematoxylin and eosin using standard methods.

### 2.5. Real-Time PCR Analysis

Approximately 50 HASTR/ci35 spheroids were collected on day 2 from the MC medium or U-bottom plate. The cells in the monolayer culture were also collected. Total RNA was extracted using the RNeasy micro kit (Qiagen, Hilden, Germany) or TRI reagent (Sigma) and reverse-transcribed into cDNA using ReverTra Ace qPCR RT Master Mix (TOYOBO, Osaka, Japan). Real-time PCR was performed with the SYBR green system (Bio-Rad Laboratories, Hercules, CA, USA) using a StepOnePlus instrument (Thermo). The target gene expression level was normalized to that of GAPDH. Primer sequences are listed in Table S1.

### 2.6. Stimulation with Inflammatory Cytokines

The 1-day-culture spheroids in the MC medium or U-bottom plate were collected and put into each well of a 6-well ultra-low attachment plate (Corning, Corning, NY, USA) at a concentration of 50 pieces/well in culture medium. The suspension culture was maintained at 37 °C under 5% CO_2_ for 72 h with TNF-α (50 ng/mL) or IL-1β (10 ng/mL) stimulation.

### 2.7. Neurite Growth Assay

The Lund human mesencephalic (LUHMES) cell line was obtained from the American Type Culture Collection. For their proliferation, we used the proliferation medium: advanced DMEM/F12 (Thermo) supplemented with alanyl-L-glutamine (2 mM, FUJIFILM Wako), N2 supplement (FUJIFILM Wako), b-FGF (40 ng/mL, FUJIFILM Wako). We established GFP-labeled LUHMES cells (GFP-luhmes) by lentiviral gene transfer. The lentivirus was produced by co-transfection of pLV-EGFP-puro (EGFP cloned VB180902-1049nfn, VectorBuilder, Chicago, IL, USA), pCMV-VSV-G-RSV-Rev (RIKEN BRC, Ibaraki, Japan), and pCAG-HIVgp (RIKEN BRC) into 293T cells (RCB2202, RIKEN BRC), using a standard protocol. GFP-luhmes were seeded at the bottom of a 6-well plate and differentiated by changing the culture medium to the differentiation medium for LUHMES cells that was the proliferation medium with some additives, db-cAMP (1 mM, Santa Cruz Biotechnology, Dallas, TX, USA), tetracycline (1 μg/mL, Sigma), glial cell line-derived neurotrophic factor (2 ng/mL, FUJIFILM Wako). After 48 h of differentiation peirod, 30 astrocytic spheroids (180,000 cells, MC-free) or monolayer cells (180,000 cells) were inoculated on the top side of the 6-well cell culture insert (Corning) and co-cultured with neuronal cells. Both top of the insert membrane and bottom of the culture plate was filled with the differentiation medium for LUHMES cells. The growth of the neurites was observed with a high-content imaging analyzer CQ1 (Yokogawa Electric Corporation, Tokyo, Japan). Image analysis was performed using the image analysis software CellPathFinder (Yokogawa). Four sets of data were acquired and the mean and standard deviation values for neurite outgrowth were calculated and graphed.

## 3. Results and Discussion

### 3.1. Rapid Formation of Astrocyte Spheroid in the MC Medium

**Figure 1:**
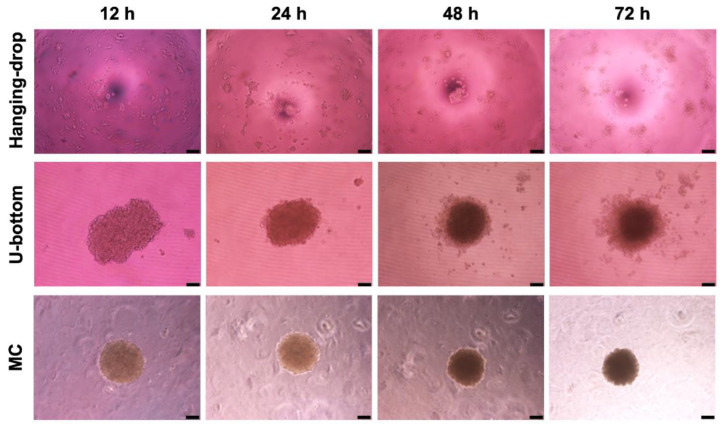
Rapid formation of HASTR/ci35 spheroids in methylcellulose medium (MC). Morphological changes in cell aggregates (6000 cells) in three different spheroid formation techniques (hanging drop, U-bottom plate, and 3% MC). Using MC medium, the spheroids were stably formed with circular shape and smooth surfaces within 12 h, the U-bottom plate method formed the aggregates but not stable, and the hanging-drop method did not form the spheroid even after 72 h. The scale bar represents 100 µm.

Immortalized human astrocytes, HASTR/ci35, are unique cell lines that retain infinite proliferation capacity when cultured at low temperature (33 °C) and are therefore a potent research source [13]. HASTR/ci35 can show relatively differentiated characteristics at 37 °C and FBS-free condition [14]. However, the degree of differentiation is still insufficient. The 3D culture is thought to be a good strategy to enhance functionality due to compaction of cells with higher cell density, leading to enhancement of cell–cell contact. In our spheroid formation method using a viscous MC medium, the cells are gathered rapidly by injecting of the cell suspension to the MC medium. Gathered cells then biologically adhere to each other and form stable 3D spheroids typically in 1 to 2 days [16,17]. Here, when HASTR/ci35 cells were injected into the MC medium, they formed aggregates in half an hour and solid spheroids were formed within 2 days (Figure 1). In contrast, the conventional low-attachment U-bottom plate formed spheroids with rough surfaces, and the hanging-drop method did not form a spheroid. These results show that HASTR/ci35 cells have relatively lower cell–cell adhesion properties because the hanging-drop method did not work well. Compared to hanging-drop method, U-bottom plate method was relatively effective to form cell aggregates. As time passes, however, the peripheral area of the aggregates became unstable, and many cells scattered away from the aggregates. It means that the MC method is the only one to form stable spheroids in these three methods. The MC method might have potential preventing diffusion of cells from the surface of the HASTR/ci35 aggregates by surrounding the entire spheroid body with a highly viscous medium.

### 3.2. Histological Analysis of HLC Spheroid

**Figure 2:**
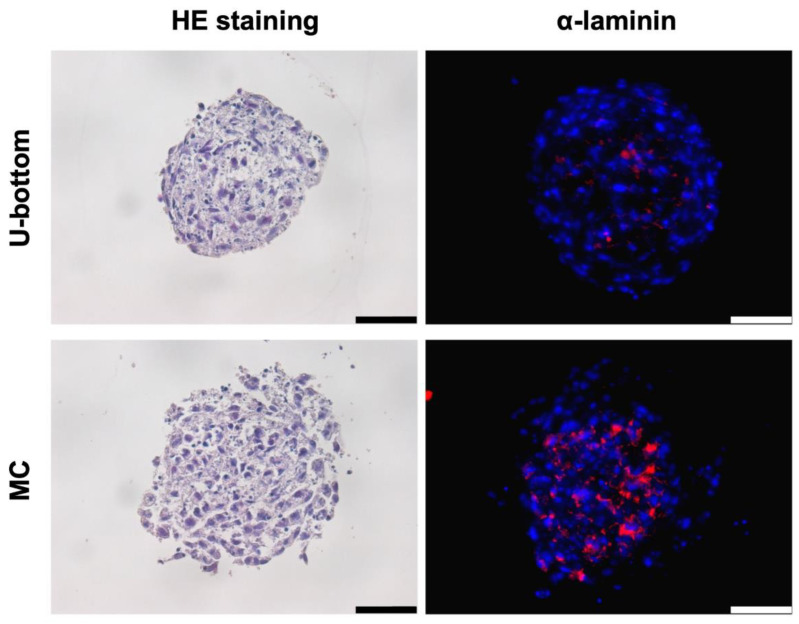
Structural and internal organization of the spheroid. Sections of spheroids were stained with hematoxylin and eosin (**left**) and immunohistochemistry (**right**). The spheroid formed and cultured in 3% MC for 72 h accumulated laminin (red) inside, whereas laminin was sparsely deposited in the spheroid formed by the U-bottom plate method. The cell nuclei were stained with Hoechst 33,342 (blue). The scale bar represents 50 µm.

To compare the morphology of the spheroids between the MC and U-bottom plate, histological patterns of extracellular matrices were examined. HE staining showed that spheroid formed in the MC methods had small gaps between the cells (Figure 2). There were no signs of necrotic cores in both the U-bottom and the MC methods. By immunohistochemical staining, laminin deposition was observed in the inner area of the 3D spheroids, especially in the MC method (Figure 2). Laminin is known to be associated with astrocyte morphogenesis, such as the end-feet process in the blood–brain barrier, and development or injury in the brain [20,21,22]. In addition, laminin affects the morphological features of astrocytes in vitro [23]. In the MC method, the spheroids were leading to enhancement of laminin deposition, and this might affect the HASTR/ci35 differentiation. Efficient accumulation of laminin may be caused by spheroid preparation by the MC method. This is because the 3% methylcellulose medium used in the MC method suppresses the free diffusion of molecules with a molecular weight of approximately 250,000 [24]. Laminin has a molecular weight of approximately 400,000 to 900,000 and may have been inhibited from diffusing into the medium [25,26]. In addition, extracellular matrices other than laminin may have accumulated inside the spheroids as well because astrocytes secrete laminin and various ECMs [27,28].

### 3.3. Gene Expression Enhancement of 3D HASTR/ci35 Spheroids

**Figure 3:**
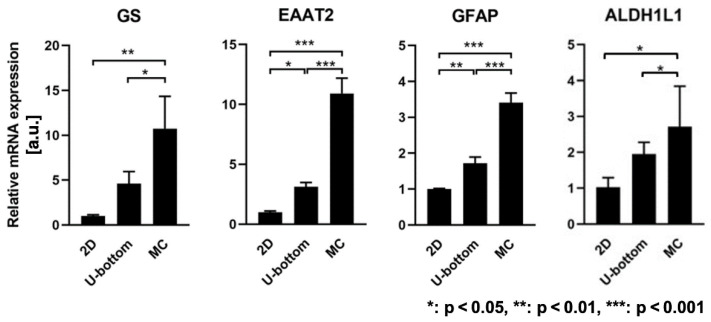
Gene expression analysis of astrocytic maturation in 3D spheroids. Expression of astrocytic gene markers was significantly enhanced in HASTR/ci35 spheroids formed using the MC method. Spheroids under both conditions were cultured for 48 h. The data are presented as mean ± SD, n = 3. *: *p*  <  0.5, **: *p*  <  0.01, ***: *p*  <  0.001, using the unpaired two-tailed Student’s *t*-test among three conditions (2D, U-bottom plate, and MC).

To examine whether the spheroid would functionally differentiate, comparative analysis of gene expression of astrocytic differentiation markers was performed between the 3D spheroids collected from the MC medium and U-bottom plate and 2D monolayer cells. We chose four differentiation markers, glutamine synthetase (GS), excitatory amino acid transporter 2 (EAAT2), glial fibrillary acidic protein (GFAP), and aldehyde dehydrogenase 1 family member L1 (ALDH1L1) [14,29]. We chose GAPDH as an internal control according to our previous work [14]. As shown in Figure 3, the results revealed that the expression levels of all differentiation markers were higher in spheroids prepared by the U-bottom method or the MC method than in 2D culture. Regarding GS and EAAT2, the expression levels in spheroids prepared by the U-bottom method were 3 to 5 times higher than those in 2D culture. On the other hand, the expression levels of GS and EAAT2 in spheroids fabricated by the MC method dramatically increased about 10-fold compared to 2D culture. The expression of GFAP and ALDH1L1 in the spheroids produced by the U-bottom method was about 2 times higher than that in the 2D state. In addition, even in spheroids formed by the MC method, the expression level remained approximately 3-fold that of the 2D culture. The induction levels of GFAP and ALDH1L1 were lower than those of GS and EAAT2, but there was a significant difference between spheroids and 2D culture conditions. GFAP is known as an astrocyte differentiation marker, but it is also a hallmark of activated astrocytes [30,31,32]. Placone et al. have reported that the switching of quiescent or active astrocytes in 3D matrices depends on the composition of extracellular matrices, such as collagen, hyaluronic acid, and Matrigel, through mechanical tension or stiffness [33]. Because laminin was accumulated in the spheroids formed with the MC method, the stiffness might be affected the expression of GFAP. We need more rigorous investigation to discuss whether the astrocytes in the spheroids are in an activated or quiescent state.

### 3.4. Inflammatory Reaction of 3D HASTR/ci35 Spheroids

**Figure 4:**
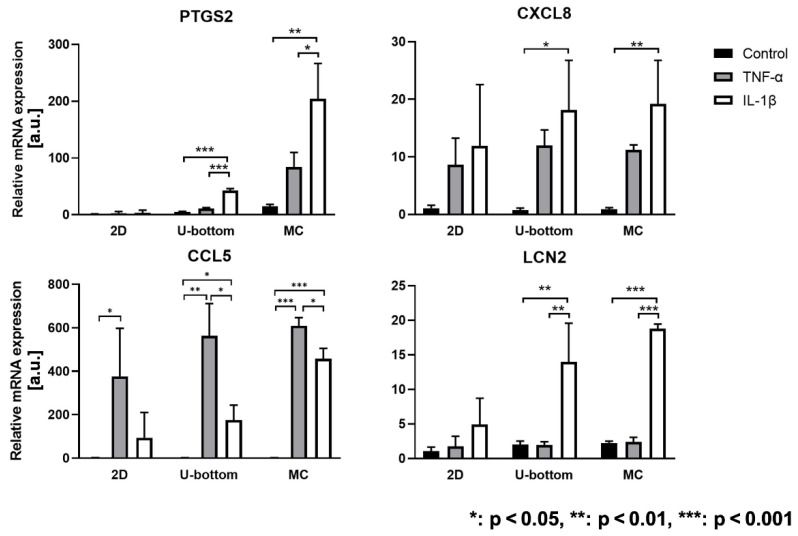
Inflammatory response of 3D spheroid cultures from astrocytes. Expression of inflammatory response genes was evaluated after stimulation with the proinflammatory cytokines TNF-α (50 ng/mL) and IL-1β (10 ng/mL) for 72 h. MC spheroids were more sensitive to cytokine stimulation, particularly PTGS2/COX2 against TNF-α and IL-1β and CCL5 against IL-1β. The data are presented as mean ± SD, n = 3. *: *p*  <  0.5, **: *p*  <  0.01, ***: *p*  <  0.001, using the unpaired two-tailed Student’s *t*-test among three conditions (control, TNF-α, and IL-1β).

Astrocytes are known to be the effector cells in inflammation [34,35,36]. To elucidate the activation potency of 3D spheroid cultures from astrocytes, the expression of inflammatory response genes was evaluated when the spheroid was stimulated with or without proinflammatory cytokines (TNF-α and IL-1β). The inflammatory related genes were selected as our previous research [14]. HASTR/ci35 cells formed spheroids by the U-bottom method or the MC method and incubated for 24 h. Spheroids were then transferred into 6-well ultra-low attachment plates and stimulated with TNF-α or IL-1β for 72 h. Figure 4 depicts that PTGS2 expression was obviously enhanced in the MC spheroids condition even the base line of the control condition was also slightly elevated. Compared to the U-bottom method, CCL5 expression was highly induced against IL-1β stimulation in the MC spheroids, but there was no difference between the U-bottom and the MC methods when they were stimulated with TNF-α. The expression of both CXCL8 and LCN2 in the spheroids formed by the MC method showed almost the same response as those of the U-bottom method. It was restrictive of the enhancement of the inflammatory reaction in the spheroids fabricated with the MC medium. The induction of PTGS2, however, was dramatically increased and it might be helpful to study the inflammatory event related with PTGS2. Although this is not an MC medium condition-specific event, LCN2 expression was only induced upon IL-1β stimulation when the cells were in a spheroid state. Astrocyte-derived LCN2 is involved in chronic itch [37], cerebral stroke [38,39] and so on. To reveal the role of LCN2 in these diseases, IL-1β-dependent and TNF-α independent induction mechanism of the of HASTR/ci35 spheroids might be a useful tool. We discussed that we could not decide whether HASTR/ci35 cells in spheroid culture were in reactive state or not. LCN2 is known as secreted from the reactive astrocytes [40]. The result of LCN2 expression in Figure 4 support they did not show their reactive phenotype.

### 3.5. Effect of 3D Astrocytes on Neurite Growth

**Figure 5:**
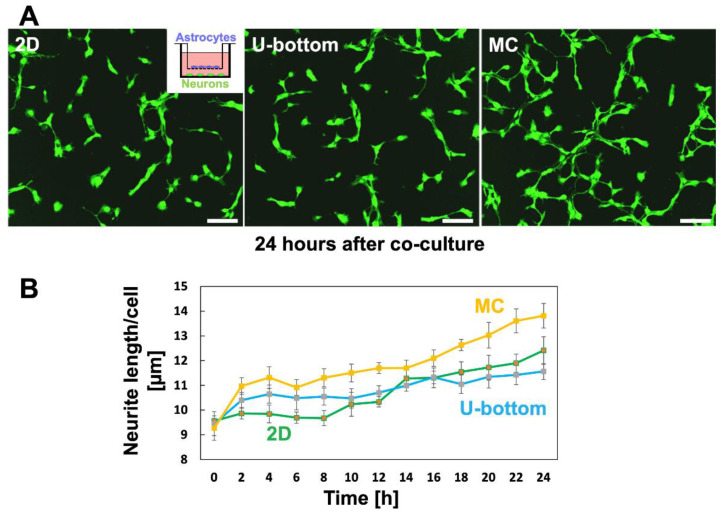
Effect of 3D spheroid cultures from astrocytes on neurite growth. Time-lapse image analysis of neurite growth of GFP-luhmes was performed. The GFP-luhmes were indirectly co-cultured with astrocyte spheroids or monolayer cells seeded on the top of the insert membrane, and neurite growth was monitored for 24 h using high-content image analyzer CQ1 The scale bar represents 100 µm (**A**). Neurite outgrowth was measured using the CellPathFinder software. Mean and standard deviation values were calculated from the four data (n = 4) (**B**).

Mature astrocytes acquire a clearance function to eliminate neurotoxic substances, such as glutamate [3,4] and secrete lactate as an energy substrate [5,6,7]. Figure 3 shows that the 3D spheroids from the MC medium enhanced the expression of glutamate transporter (EAAT2) and GS, which synthesize glutamine from glutamate, assuming that it controls neuronal substances in the culture medium. To clarify the effect of this clearance, an in vitro co-culture model using cell culture inserts and time-lapse image analysis was performed. Astrocyte spheroids or monolayer cells seeded on the top of the insert membrane were indirectly in contact with GFP-expressing neuronal cells (GFP-luhmes) at the bottom of the culture plate. The GFP-luhmes were cultured for 24 h with 2D or 3D cultured astrocytes at 37 °C and 5% CO_2_, and the fluorescence images were acquired using a high-content image analyzer CQ1 (Yokogawa) every 2 h. Image analysis was performed using the protocol implemented in the image analysis software. As shown in Figure 5, co-culture with 2D astrocytes regained neurite growth to some extent during the second half of the day, and protrusions of the neurons were often observed. When 3D spheroids were cultured on the insert membrane, neurite growth was initially accelerated, and the growth was remarkable in the condition using spheroids from the MC medium, indicating that the ability of astrocytes to support neurite growth was correlated with their maturation states. Unfortunately, the student’s *t*-test showed no significant difference between the MC method and the U-bottom method or the 2D condition, except for the data at 22 h. However, the spheroids formed with the MC method exhibited the trend of superiority in supporting neuronal cell growth over the entire culture period. The co-culture of various combinations of astrocytes and neurons are reported. Astrocytes influence neurite outgrowth not only in direct co-culture [41], but also in indirect co-culture with culture inserts [13,42]. The fact that different spheroid formation methods have different neuroprotective effects is a remarkable result as 3D culture becomes more popular in the future.

Throughout this study, HASTR/ci35 spheroids formed by the MC medium acquired the differentiation phenotype, exhibited inflammatory responses, and supported neurite growth, indicating that 3D spheroid cultures using astrocytes from the MC medium were a potent model for mature astrocytes. We believed that one of the direct reasons of these phenomena was ECM accumulation by the MC method. In addition to cell–cell communications by stable spheroid formation, cell–ECM interactions might enhance the differentiational functions. Of course, the spheroid culture is not the best culture method for the astrocytes. Developing more physiologically normal culture methods would help for further astrocyte functioning in future. For example, blood stream with vessels as well as neurons and other components in the brain should be equipped to the 3D culture. Meanwhile, this technique could be useful for culturing immortalized astrocytes, or astrocyte-like cells derived from various stem cells.

## 4. Conclusions

Our spheroid formation method realized the solid formation of a human astrocyte spheroid with a well-differentiated phenotype, capability for inflammation, and supportive potency for neurons. We believe that our 3D technique uniquely can accelerate the functional enhancement and biomimeticity of the 3D spheroid model. Especially, ECM deposition from the astrocytes would affect other types of brain cells. Hetero spheroid formation with neurons and/or endothelial cells by the MC method is the next challenge to reveal the mechanisms of cell–cell and cell–ECM interactions.

## Data Availability

Please contact us at any time if you have any questions about the data.

## Supplementary Materials

The following supporting information can be downloaded at: https://www.mdpi.com/article/10.3390/bioengineering10030349/s1, Table S1: Primers used in real-time PCR analysis.

## References

[1] Khakh B.S., Sofroniew M.V. (2015). Diversity of astrocyte functions and phenotypes in neural circuits. Nat. Neurosci..

[2] Singh A.V., Chandrasekar V., Laux P., Luch A., Dakua S.P., Zamboni P., Shelar A., Yang Y., Pandit V., Tisato V. (2022). Micropatterned Neurovascular Interface to Mimic the Blood-Brain Barrier’s Neurophysiology and Micromechanical Function: A BBB-on-CHIP Model. Cells.

[3] Goubard V., Fino E., Venance L. (2011). Contribution of astrocytic glutamate and GABA uptake to corticostriatal information processing. J. Physiol..

[4] Schousboe A., Bak L.K., Waagepetersen H.S. (2013). Astrocytic Control of Biosynthesis and Turnover of the Neurotransmitters Glutamate and GABA. Front. Endocrinol..

[5] Dienel G.A. (2017). Lack of appropriate stoichiometry: Strong evidence against an energetically important astrocyte-neuron lactate shuttle in brain. J. Neurosci. Res..

[6] Magistretti P.J., Allaman I. (2018). Lactate in the brain: From metabolic end-product to signalling molecule. Nat. Rev. Neurosci..

[7] Karagiannis A., Gallopin T., Lacroix A., Plaisier F., Piquet J., Geoffroy H., Hepp R., Naude J., Le Gac B., Egger R. (2021). Lactate is an energy substrate for rodent cortical neurons and enhances their firing activity. eLife.

[8] Sloan S.A., Darmanis S., Huber N., Khan T.A., Birey F., Caneda C., Reimer R., Quake S.R., Barres B.A., Pasca S.P. (2017). Human Astrocyte Maturation Captured in 3D Cerebral Cortical Spheroids Derived from Pluripotent Stem Cells. Neuron.

[9] Roybon L., Lamas N.J., Garcia A.D., Yang E.J., Sattler R., Lewis V.J., Kim Y.A., Kachel C.A., Rothstein J.D., Przedborski S. (2013). Human stem cell-derived spinal cord astrocytes with defined mature or reactive phenotypes. Cell Rep..

[10] Zhou B., Zuo Y.X., Jiang R.T. (2019). Astrocyte morphology: Diversity, plasticity, and role in neurological diseases. CNS Neurosci. Ther..

[11] Khakh B.S., Deneen B. (2019). The Emerging Nature of Astrocyte Diversity. Annu. Rev. Neurosci..

[12] Endo F., Kasai A., Soto J.S., Yu X., Qu Z., Hashimoto H., Gradinaru V., Kawaguchi R., Khakh B.S. (2022). Molecular basis of astrocyte diversity and morphology across the CNS in health and disease. Science.

[13] Furihata T., Ito R., Kamiichi A., Saito K., Chiba K. (2016). Establishment and characterization of a new conditionally immortalized human astrocyte cell line. J. Neurochem..

[14] Kitamura K., Ito R., Umehara K., Morio H., Saito K., Suzuki S., Hashimoto M., Saito Y., Anzai N., Akita H. (2018). Differentiated HASTR/ci35 cells: A promising in vitro human astrocyte model for facilitating CNS drug development studies. J. Pharmacol. Sci..

[15] Urich E., Patsch C., Aigner S., Graf M., Iacone R., Freskgard P.O. (2013). Multicellular self-assembled spheroidal model of the blood brain barrier. Sci. Rep..

[16] Kojima N., Takeuchi S., Sakai Y. (2012). Rapid aggregation of heterogeneous cells and multiple-sized microspheres in methylcellulose medium. Biomaterials.

[17] Tao F., Mihara H., Kojima N. (2019). Generation of Hepatic Tissue Structures Using Multicellular Spheroid Culture. Methods Mol. Biol..

[18] Sayo K., Aoki S., Kojima N. (2016). Fabrication of bone marrow-like tissue in vitro from dispersed-state bone marrow cells. Regen. Ther..

[19] Tao F., Hanada S., Matsushima K., Arakawa H., Ishida N., Kato Y., Okimura S., Watanabe T., Kojima N. (2023). Enhancement and maintenance of hepatic metabolic functions by controlling 3D aggregation of cryopreserved human iPS cell-derived hepatocyte-like cells. J. Biosci. Bioeng..

[20] Ichikawa-Tomikawa N., Ogawa J., Douet V., Xu Z., Kamikubo Y., Sakurai T., Kohsaka S., Chiba H., Hattori N., Yamada Y. (2012). Laminin alpha1 is essential for mouse cerebellar development. Matrix Biol..

[21] Menezes M.J., McClenahan F.K., Leiton C.V., Aranmolate A., Shan X., Colognato H. (2014). The extracellular matrix protein laminin alpha2 regulates the maturation and function of the blood-brain barrier. J. Neurosci..

[22] Zapata-Acevedo J.F., Garcia-Perez V., Cabezas-Perez R., Losada-Barragan M., Vargas-Sanchez K., Gonzalez-Reyes R.E. (2022). Laminin as a Biomarker of Blood-Brain Barrier Disruption under Neuroinflammation: A Systematic Review. Int. J. Mol. Sci..

[23] Sato J., Horibe S., Kawauchi S., Sasaki N., Hirata K.I., Rikitake Y. (2018). Involvement of aquaporin-4 in laminin-enhanced process formation of mouse astrocytes in 2D culture: Roles of dystroglycan and alpha-syntrophin in aquaporin-4 expression. J. Neurochem..

[24] Tao F., Sayo K., Sugimoto K., Aoki S., Kojima N. (2020). Development of a tunable method to generate various three-dimensional microstructures by replenishing macromolecules such as extracellular matrix components and polysaccharides. Sci. Rep..

[25] Aumailley M., Bruckner-Tuderman L., Carter W.G., Deutzmann R., Edgar D., Ekblom P., Engel J., Engvall E., Hohenester E., Jones J.C. (2005). A simplified laminin nomenclature. Matrix Biol..

[26] Aumailley M. (2013). The laminin family. Cell Adhes. Migr..

[27] Yao Y., Chen Z.L., Norris E.H., Strickland S. (2014). Astrocytic laminin regulates pericyte differentiation and maintains blood brain barrier integrity. Nat. Commun..

[28] Allnoch L., Leitzen E., Zdora I., Baumgartner W., Hansmann F. (2022). Astrocyte depletion alters extracellular matrix composition in the demyelinating phase of Theiler’s murine encephalomyelitis. PLoS ONE.

[29] Jurga A.M., Paleczna M., Kadluczka J., Kuter K.Z. (2021). Beyond the GFAP-Astrocyte Protein Markers in the Brain. Biomolecules.

[30] Pekny M., Wilhelmsson U., Tatlisumak T., Pekna M. (2019). Astrocyte activation and reactive gliosis-A new target in stroke?. Neurosci. Lett..

[31] Giovannoni F., Quintana F.J. (2020). The Role of Astrocytes in CNS Inflammation. Trends Immunol..

[32] Brenner M., Messing A. (2021). Regulation of GFAP Expression. ASN Neuro.

[33] Placone A.L., McGuiggan P.M., Bergles D.E., Guerrero-Cazares H., Quinones-Hinojosa A., Searson P.C. (2015). Human astrocytes develop physiological morphology and remain quiescent in a novel 3D matrix. Biomaterials.

[34] Sofroniew M.V. (2014). Multiple roles for astrocytes as effectors of cytokines and inflammatory mediators. Neuroscientist.

[35] Sofroniew M.V. (2015). Astrocyte barriers to neurotoxic inflammation. Nat. Rev. Neurosci..

[36] Linnerbauer M., Wheeler M.A., Quintana F.J. (2020). Astrocyte Crosstalk in CNS Inflammation. Neuron.

[37] Shiratori-Hayashi M., Tsuda M. (2020). Role of reactive astrocytes in the spinal dorsal horn under chronic itch conditions. J. Pharmacol. Sci..

[38] Luo C., Zhou S., Yin S., Jian L., Luo P., Dong J., Liu E. (2022). Lipocalin-2 and Cerebral Stroke. Front. Mol. Neurosci..

[39] Zhao R.Y., Wei P.J., Sun X., Zhang D.H., He Q.Y., Liu J., Chang J.L., Yang Y., Guo Z.N. (2023). Role of lipocalin 2 in stroke. Neurobiol. Dis..

[40] Bi F., Huang C., Tong J., Qiu G., Huang B., Wu Q., Li F., Xu Z., Bowser R., Xia X.G. (2013). Reactive astrocytes secrete lcn2 to promote neuron death. Proc. Natl. Acad. Sci. USA.

[41] Oh H.N., Park S., Lee S., Chun H.S., Shin W.H., Kim W.K. (2022). In vitro neurotoxicity evaluation of biocidal disinfectants in a human neuron-astrocyte co-culture model. Toxicol. Vitr..

[42] Zhao J., Gonsalvez G.B., Mysona B.A., Smith S.B., Bollinger K.E. (2022). Sigma 1 Receptor Contributes to Astrocyte-Mediated Retinal Ganglion Cell Protection. Investig. Investig. Opthalmol. Vis. Sci..

