# Supplementary material for: Rapid and Stable Formation Method of Human Astrocyte Spheroid in a High Viscous Methylcellulose Medium and Its Functional Advantages
Source: Bioengineering (Basel). 2023 Mar 11;10(3):349. doi: 10.3390/bioengineering10030349 (PMC10045153; doi:10.3390/bioengineering10030349)
Supplement: Supplementary file 1 [file bioengineering-10-00349-s001.zip › bioengineering-1982783-supplementary.pdf]

Table S1. Primers used in real-time PCR analysis.

Primer name[a]	Sequence (5’ > 3’)
GAPDH F	TGC ACC ACC AAC TGC TTA
GAPDH R	GGA TGC AGG GAT GAT GTT C
GS F	AAG AGC GGA GCG TGT GAG
GS R	TCA TGG TGG AAG GTG TTC TG
EAAT2 F	TCT CCC CAG TAC CTC TCC AG
EAAT2 R	TGG GCA TAT TGT TGG CAC T
GFAP F	ACA GGA AGC TGC TAG AGG GCG A
GFAP R	CCA GGC TGG TTT CTC GAA TCT G
ALDH1L1 F	ATC ACC CGT CAC TGC TCC CTA
ALDH1L1 R	CCC CCC TTT CTT ATC TCC GTG
PTGS2 F	CTT CAC GCA TCA GTT TTT CAA G
PTGS2 R	TCA CCG TAA ATA TGA TTT AAG TCC AC
CCL5 F	TGC CCA CAT CAA GGA GTA TTT
CCL5 R	CTT TCG GGT GAC AAA GAC G
CXCL8 F	GAG CAC TCC ATA AGG CAC AAA
CXCL8 R	ATG GTT CCT TCC GGT GGT
LCN2 F	CTC CAC CTC AGA CCT GAT CC
LCN2 R	ACA TAC CAC TTC CCC TGG AAT

[a], F and R indicate forward and reverse direction.
